# Deep Learning Prediction of Cancer Prevalence from Satellite Imagery

**DOI:** 10.3390/cancers12123844

**Published:** 2020-12-19

**Authors:** Jean-Emmanuel Bibault, Maxime Bassenne, Hongyi Ren, Lei Xing

**Affiliations:** 1 Laboratory of Artificial Intelligence in Medicine and Biomedical Physics, Stanford University School of Medicine, Stanford, CA 94304, USA; bassenne@stanford.edu (M.B.); hongyi@stanford.edu (H.R.); 2 Radiation Oncology Department, Hôpital Européen Georges Pompidou, Assistance Publique–Hôpitaux de Paris, 75015 Paris, France

**Keywords:** cancer, epidemiology, deep learning

## Abstract

**Simple Summary:**

Cancer prevalence estimates are used to guide policymaking, from prevention to screening programs. However, these data are only available for 28% of the U.S. population. We used deep learning to analyze satellite imagery in order to predict cancer prevalence with a high spatial resolution. This method explained up to 64.37% of the variation of cancer prevalence. It could potentially be used to map cancer prevalence in entire regions for which these estimates are currently unavailable.

**Abstract:**

The worldwide growth of cancer incidence can be explained in part by changes in the prevalence and distribution of risk factors. There are geographical gaps in the estimates of cancer prevalence, which could be filled with innovative methods. We used deep learning (DL) features extracted from satellite images to predict cancer prevalence at the census tract level in seven cities in the United States. We trained the model using detailed cancer prevalence estimates from 2018 available in the CDC (Center for Disease Control) 500 Cities project. Data from 3500 census tracts covering 14,483,366 inhabitants were included. Features were extracted from 170,210 satellite images with deep learning. This method explained up to 64.37% (median = 43.53%) of the variation of cancer prevalence. Satellite features are highly correlated with individual socioeconomic and health measures that are linked to cancer prevalence (age, smoking and drinking status, and obesity). A higher similarity between two environments is associated with better generalization of the model (*p* = 1.10–6). This method can be used to accurately estimate cancer prevalence at a high spatial resolution without using surveys at a fraction of the cost.

## 1. Introduction

Accurate and detailed measures of cancer prevalence are decisive factors driving research and policymaking: they are required to better understand the link between environment and cancer, to discover population clusters, and to adapt the healthcare capacity. The coverage and granularity of these estimations are currently limited. The World Health Organization publishes these measures in the GLOBOCAN (Global Cancer Observatory) estimates of incidence and mortality, at the scale of a country or region [1]. In the United States, the American Community Survey (ACS), which is performed by the Census Bureau and cost USD 220 million in 2018 [2], provides demographical data for all cities and counties with a population of 65,000 or more [3]. For smaller regions, cancer incidence reports have been shown to be unreliable [4]. Several other programs specifically document cancer epidemiology: The National Cancer Institute’s Surveillance, Epidemiology, and End Results (SEER) Program collects and publishes cancer incidence and survival data from population-based cancer registries that cover approximately 28% of the U.S. population [5]. Furthermore, the National Program of Cancer Registries provides data on cancer at the scale of a state. Changing population exposure to modifiable risk factors is a key driver of cancer prevention. Having detailed estimates of the geographical distribution of cancer is therefore important when optimizing risk-reduction policies. Curating the information to fill these gaps on a national scale would require a sharp financial and human effort. Considering these difficulties, new methods using readily available data are required to provide detailed and accurate estimates. Novel sources of passively collected data are available today: an approach was recently described to leverage satellite images in order to explore different socio-demographic or health outcomes by correlating luminosity at night with economic activity [6], or built environment with obesity [7]. Here, we adapt these methods to create a high-resolution predictive model of the spatial distribution of cancer prevalence within seven of the most-populated cities in the United States.

## 2. Results

### 2.1. City Characteristics and Feature Extraction

**Figure 1:**
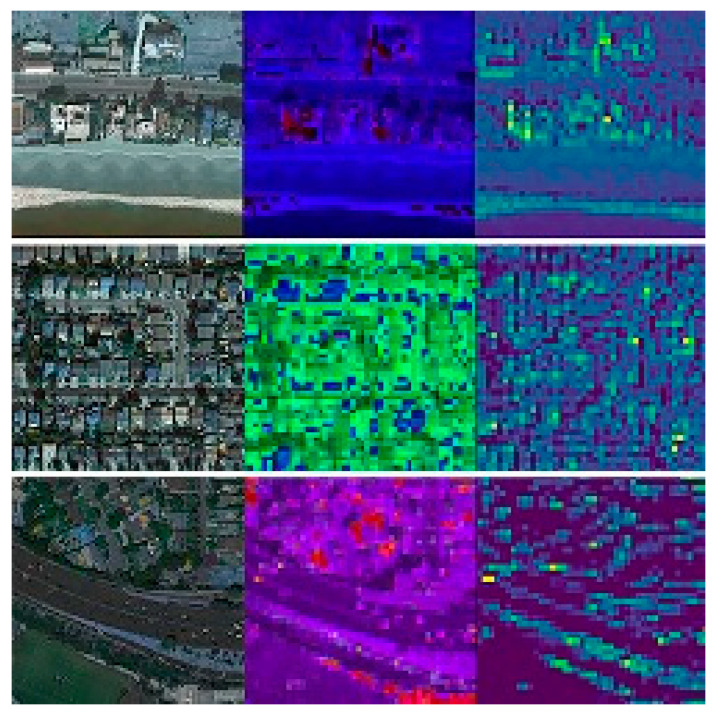
Visualization of features from the activation maps from the first and second residual blocks of ResNet50.

In total, 3500 census tracts (18,000 km^2^—6979.2 square miles) with 14,483,366 inhabitants were included. The actual average cancer prevalence was 5% in Chicago (*n* = 2,705,994), 4.8% in Dallas (*n* = 1,345,047), 4.8% in Houston (*n* = 2,325,502), 4.9% in Los Angeles (*n* = 3,990,456), 5.4% in Phoenix (*n* = 1,660,272), 5% in San Diego (*n* = 1,425,976), and 4.9% in San Jose (*n* = 1,030,119). Features from the activation maps from the first and second residual block of ResNet50 are shown in Figure 1.

To verify the capacity of the neural network to extract relevant features from satellite images, we generated a grid t-SNE (t-distributed Stochastic Neighbor Embedding) that clusters a random subset of 1000 images from each city in an unsupervised manner (Figure S1A–G).

### 2.2. Optimization of ElasticNet

**Figure 2:**
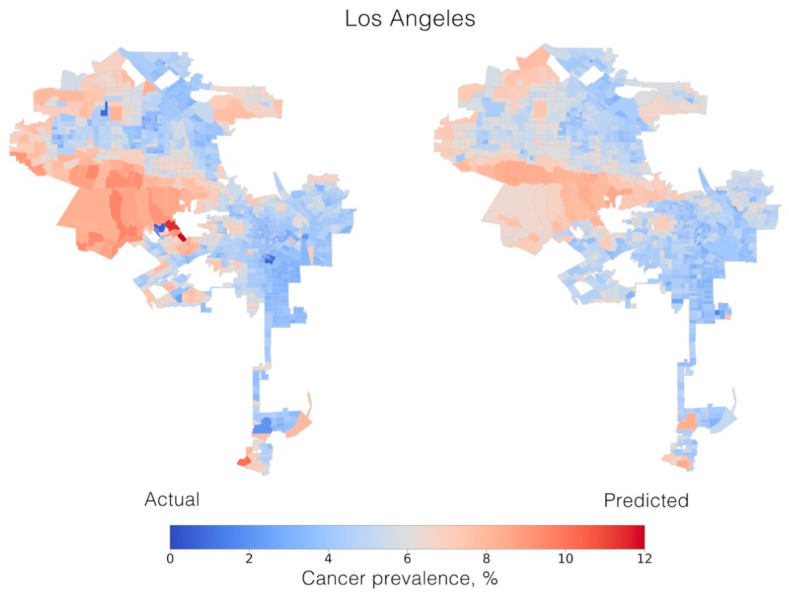
Maps of actual (**left**) and predicted (**right**) cancer prevalence in Los Angeles, CA, USA, at the census-tract level.

The hyperparameters were tuned using grid search: in total, 298,500 runs were performed for each model, fitting five folds for each of the 59,700 candidate models. In each individual city, the satellite features explained between 40.19 (±0.23) and 64.37% (±6.05%) of the variation in cancer prevalence in out-of-sample estimates (MSE = 0.96 ± 0.23; MAE = 0.73 ± 0.07), proving that this approach can accurately predict cancer prevalence using satellite images alone (Figure 2 and Figure S2A–E).

Complete results with 95% confidence intervals are provided in Table 1.

We illustrate the relationship between predicted and actual cancer prevalence in each individual census across all cities in Figure S3.

### 2.3. Model Generalization and Environment Similarities

We assessed the performance of a model trained on one city and used it to predict cancer prevalence from the satellite images from the other cities. In parallel, we calculated the satellite feature similarity between each city by calculating the cosine similarity in a pair-wise manner. For example, a model created with the data from Los Angeles was used to predict cancer prevalence in all the other cities from our dataset. A higher similarity between two environments is significantly associated with better estimation performances, while a greater dissimilarity will decrease the predictive performances (Figure S4: Correlation coefficient between performance and cosine similarity = 0.48, *p* = 1.10^−6^).

### 2.4. Model Interpretability

By fitting the individual socioeconomic and health measures from the ACS and the CDC to the extracted deep learning features, we measured the strength of the relationship between the features and the measures. Overall, these features, extracted with ResNet50 from unlabeled satellite images, provided a median *r*^2^ score of 86% (range: 65.5–91%), suggesting that they are well representative of the determinants of cancer environmental risk factors (Table 2).

The features extracted with Deep Learning (DL) from the satellite images are closely related to socioeconomic factors, such as obesity (*r*^2^ = 91%), physical activity (*r*^2^ = 89.8%), or smoking status (*r*^2^ = 89.5%).

## 3. Discussion

The link between cancer and environment has been explored for more than 50 years [8]. Cancers, like all human disease, follow a pattern and tend to cluster within the environment on which they depend and contain their causative agents. Recognizing these clusters can be used as a guide to identify the factors responsible for their causation. Researchers have tried to find the causes of different types of cancer by using different methods: nutritional surveys were among the first studies that explicitly showed a correlation between eating habits and breast and colon cancer [9]. In 1973, Drasar et al. used data from the Food and Agriculture Organization from 37 countries and showed that animal fat and protein intake were highly correlated with these cancers. In the 1980s, environment registries were created and used to monitor specific populations [10], for example, electronics industry workers [11] or agricultural workers [12]. More recently, cohorts of twins were used to assess the contribution of hereditary vs. environmental factors to the causation of sporadic cancer cases [13]. Data from 44,788 pairs of twins listed in the Swedish, Danish, and Finnish twin registries were combined to assess the risks of cancer at 28 anatomical sites. The authors concluded that “the environment has the principal role in causing sporadic cancer”. To further study this association, surveys are regularly performed in the U.S. Since 1959, the American Cancer Society has used detailed survey questionnaires within their Epidemiology Research Program [14]. Over 60 years, data have been collected from nearly 2.7 million U.S. men and women. Other programs exploring the link between environment and cancer include the National Cancer Institute (NCI)’s SEER and the Census Bureau ACS. All these programs have collected extremely valuable data, which have allowed for examples to reveal the link between smoking and cancer [15]. These methods also have limitations, specifically because they are costly, require a significant human effort to conduct, do not cover the entire nation [5], and have a low spatial resolution when it comes to disease geographical distribution [4].

Modeling exposure to risk factors is a complex and costly task, and using satellite images that directly reflect socioeconomic status, population age, and pollutant exposure is an elegant solution. Our study is the first to leverage readily available data to cover more than 14 million inhabitants. We show that DL can be used to extract features from satellite images that explain up to 64.37% of the variations of cancer prevalence. This correlation, however, does not imply a causal link between the environment and cancer. Potential biases include socioeconomic status, such as ethnicity, education, and income, which are in large part related to where people live. 

This method was chosen because a previous study had already shown that Convolutional Neural Networks (CNN) were more suitable for this task than simpler methods, such as average pixel value of an image (RGB), color histograms, histogram of oriented gradients (HOG), or principal component analysis (PCA) [6]. Labels of geographical points of interest (POIs) have also been shown to be less effective than direct features extracted with a CNN from satellite images [7]. Environmental factors in the causation of cancer are unlikely to have changed over time from their current state of prevalence as reflected in the images.

This proof-of-concept study has several limitations. First, we were unable to measure cancer prevalence for each anatomical site, because we relied on the CDC 500 Cities project data that only provided all-cancer prevalence (except skin). We also do not have estimates for pediatric cancers or data from middle- or low-income countries, where the need for this kind of new method is the most important. Secondly, since the cancer prevalence estimates result from a multilevel logistic regressions model that uses socio-demographic features from the ACS, we cannot use these same socio-demographic descriptors as predictors to compare the performances of our method. Thirdly, we were not able to validate the robustness of the models to longitudinal changes because the data from the 500 Cities project cannot be used to track changes at the local level over time [16].

To interpret the results of our study, we should also consider that the models were trained on large cities from North America and that the results could potentially not translate to cities with very differently built environments, such as European or Asian cities. A validation using European data would have been ideal, but the required data currently do not exist. This also highlights the importance of having detailed, high resolution epidemiologic data for health outcomes.

Finally, this method uses CNNs pre-trained for natural object recognition and classification. CNNs fully trained on satellite images could potentially provide better performance but would require millions of satellite images labeled for a specific health outcome. In this study, we found that using a transfer learning approach is a reliable alternative. Genetics is an important cancer driver when considering and comparing worldwide populations. This may make these models less generalizable because people of similar genetic backgrounds may be more likely to live in geographically clustered areas, thereby affecting cancer prevalence in those areas due to reasons unrelated to the particular environment.

This type of method could be used to better assess cancer prevalence at a high spatial resolution in unsurveyed areas of only a few thousand people. These kinds of results could potentially help to detect unusual clusters of high cancer prevalence. This could lead to better public health interventions to mitigate these clusters through screening, education, and environmental adaptations. Finally, beyond the USA, middle- or low-income countries (MLIC) could benefit from the proposed method: A deep learning-supported project may enable the MLICs to collect much more targeted and modest amounts of data for the training of models, which could then be used to cost-effectively, and hopefully reliably, estimate prevalence in the rest of the country. While deep learning does not obviate the need for careful data extraction, it can help accelerate results, and, despite its data-hungry nature, help conserve strained resources.

Beyond cancer epidemiology, automated deep learning analysis of satellite images could be used to identify clusters for many different health outcomes related to environmental and socioeconomic features, including, but not limited to preterm birth as well as cardiovascular and infectious diseases. Among the major environmental hazards that could potentially be detected with this method are unsafe water, poor sanitation and hygiene, urban air pollution, and climate change. This method could be used as an opportunity to perform preemptive interventions through healthier environments.

## 4. Materials and Methods

This study was exempt from institutional review board approval because the research used existing data and records collected by external parties so that no individual could be identified. We used the Strengthening the Reporting of Observational Studies in Epidemiology (STROBE) reporting guideline where applicable [17]. All code is available on a GitHub repository at https://github.com/jebibault/CancerSatellite.

### 4.1. Cancer Prevalence Data

We used the 2018 estimates of annual crude cancer prevalence from the 500 Cities project, a collaboration between the Center for Disease Control and Prevention (CDC), the Robert Wood Johnson Foundation, and the CDC Foundation [16]. The 500 Cities covers data for the 500 largest American cities. Among these 500 cities, there are approximately 28,000 census tracts. The project includes a total population of 103,020,808, which represents 33.4% of the total United States population of 308,745,538. The 500 Cities project provides small-area estimates (6000 to 8000 inhabitants) and can be used to understand the precise geographic distribution of health-related variables and assist in planning public health interventions.

### 4.2. Satellite Imagery

We downloaded satellite images in jpg format from Google Maps at the 18 zoom level (scale ratio 1:4513) between 15 May and 16 July, 2019 [18]. These images were recorded in 2018. Each city was divided in tiles of 224 × 224 × 3 pixel RGB images. For each image file, we concomitantly generated a matching geotagging file that provided their latitudinal and longitudinal coordinates. These coordinates were used to attribute each image to their respective census using the Federal Communications Commission (FCC) Application Programming Interface (API) [19].

### 4.3. Image Features Extraction

Convolutional neural networks (CNNs) have disrupted the field of computer vision and object recognition since 2012, when AlexNet won the ImageNet Large Scale Visual Recognition Challenge [20]. CNNs trained on very large datasets with millions of natural objects have been successfully employed for tasks ranging from skin cancer recognition [21] to language modeling [22] and financial forecasting [23]. A transfer learning approach was used: a deep neural network was used to extract features of the environment from unlabeled satellite images [24]. We assessed the performances of EfficientNet B7, InceptionResNetV2, NASNetLarge, ResNet50, and VGG16. ResNet50 provided the best performance (Table S1). Characteristics of ResNet50 are available in Table S2. The network was loaded with the pre-trained weights of ImageNet. The top layer, which usually performs the classification, was then removed and the outputs were saved. To reduce dimensionality, a sample-based average pooling was performed. The Keras (v. 2.2.5) library was used to perform the feature extractions [25]. Because we have the cancer prevalence estimates at the scale of a census-tract from the 500 cities project, we need to fit the vectors from all the images belonging to a census-tract into a single vector before we can use the ElasticNet: feature vectors from each image were clustered in their respective census based on their centroid location and feature values were averaged. At the end, each census was represented by a single vector, corresponding to the mean of the features extracted from the images included in it.

To better understand the type of features extracted by the network, we saved the output after the first and second residual blocks of the CNN as images.

### 4.4. Grid t-Distributed Stochastic Neighbor Embedding (t-SNE)

To verify that the extractors form an effective representation of the environment, we postulated that the features extracted should allow us to group the images by content similarity. In order to do so, we performed a grid t-SNE for each individual city, using the features generated with the CNN from each image. Before we performed the t-SNE, we reduced dimensionality to 300 features with a principal component analysis (PCA) for two reasons: (1) the feature vectors may have some redundancy which could skew similarity comparisons towards overrepresented features and (2) such a large number of features would not be optimal for computation costs and runtime. We then reduced our image dataset to 1000 random images per city.

### 4.5. ElasticNet Regression Hyperparameters Tuning

Because previous studies described the same analysis pipeline [6,7], we used the features obtained from the extractor to create a model with a regularized regression to predict cancer prevalence from satellite images for each individual city. This step included: standardization of the variables (where we removed the mean and scaled to unit variance), selection of the best features, and nested, binned, stratified (5 splits, shuffled, with a random stat = 42), 5-fold cross-validated grid search optimization of the regularization parameters. The parameters optimized were the number of best features (range 1–200), the L1 ratio (0.05, 0.15, 0.95, 0.99, 1), and the alpha parameter (logspace[−5, 0.5, 60]). Optimization parameters included the number of features used for the prediction, a penalty term (alpha), and the L1/L2 ratio.

Metrics are reported on each city and include the *r*^2^ score, the mean squared error (MSE), and the mean absolute error (MAE) with 95% confidence intervals. 

The *r*^2^ score is the proportion of the variance in the dependent variable that is predictable from the independent variable(s). It provides a measure of how well observed outcomes are replicated by the model based on the proportion of total variation of outcomes explained by the model. A perfect model would have a score of 100%. If $\hat{y}i$ is the predicted value of the *i*th sample and yi is the corresponding true value, then the estimated *r*^2^ is defined as:

$$r^{2}=1-\frac{\sum_{i=1}^{n}{\left(yi-\hat{y}i\right)}^{2}}{\sum_{i=1}^{n}{\left(yi-\bar{y}i\right)}^{2}}.$$

The MAE is a measure of errors between paired observations. It is defined as:

$$MAE=\frac{1}{n}\;\sum_{i=1}^{n}\left|yi-\hat{y}i\right|.$$

The MSE measures the average of the squares of the errors. It is defined as:

$$MSE=\frac{1}{n}\;\sum_{i=1}^{n}{\left(yi-\hat{y}i\right)}^{2}.$$

A separate test dataset (20% of the data randomly selected from the complete dataset) was excluded from the training set and then used to perform cancer prevalence predictions and plot the map of cancer prevalence for each city.

### 4.6. Model Generalization and Environment Similarities

The distribution of environment and landscape features could potentially be different from one city to another and could limit the generalization of a model. To better understand, we computed the cosine similarity (CS) between the features extracted in a pair-wise manner. CS can be used to assess the similarity of two images: two feature vectors with the same orientation have a cosine similarity of 1, two vectors oriented at 90° relative to each other have a similarity of 0, and two vectors diametrically opposed have a similarity of −1. To assess whether or not our models could provide accurate predictions of cancer prevalence in cities on which they had not been trained, we computed the *r*^2^ score, mean squared error and mean absolute error of each of the models when used to predict cancer prevalence from data they had never seen. We then calculated the correlation between model performance and CS.

### 4.7. Model Interpretability

To provide a level of interpretability and more robust insights into the drivers of cancer prevalence, the relationships between the features extracted from the satellite images and the following population-level socioeconomic and health measures were explored:

- Socioeconomic (from the 2018 ACS):
  ○ Age
  ○ Sex (reference: female)
  ○ Race (reference: non-Hispanic white)
  ○ Educational attainment (reference: high school diploma)
  ○ Median per capita income
- Health (from the CDC 500 cities project):
  ○ Smoking status
  ○ Binge drinking status
  ○ Obesity
  ○ Diabetes
  ○ Physical activity

A linear regression was fitted between each of these measures and the satellite deep learning features for every census tract and the *r*^2^ score was calculated.

### 4.8. Software Packages

**Figure 3:**
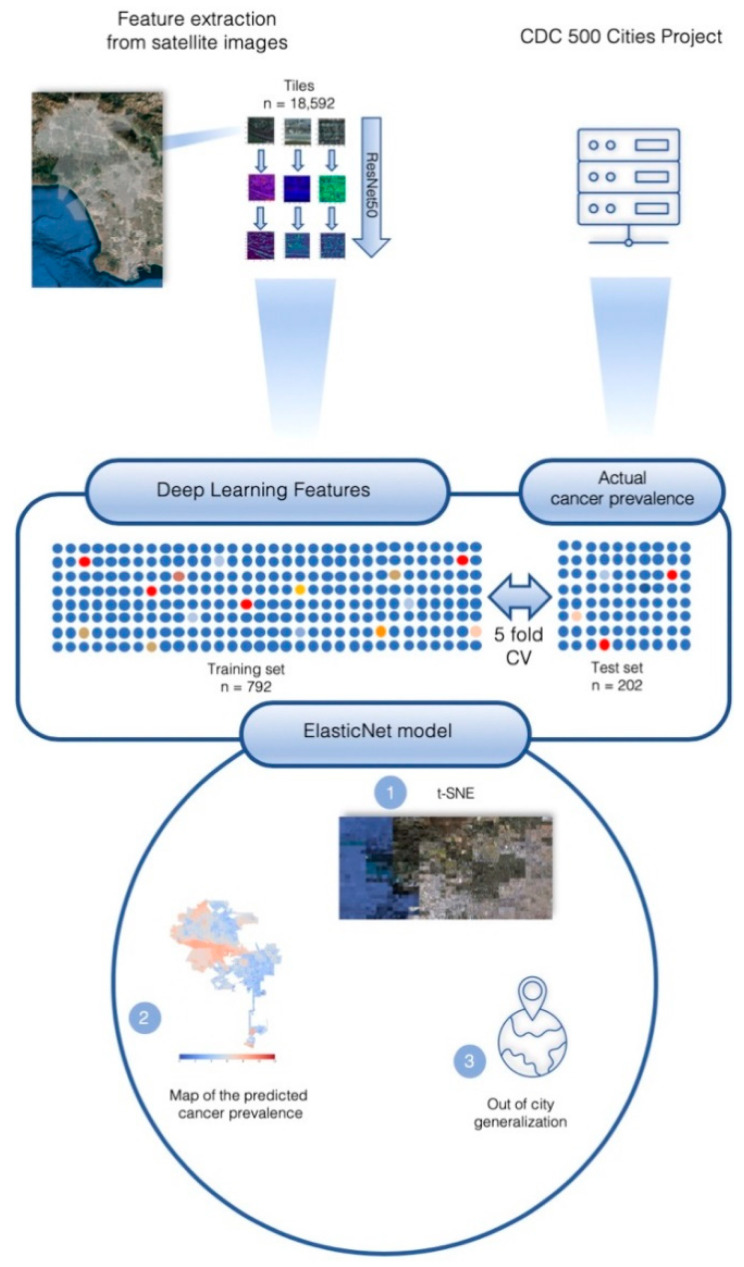
The deep learning features were extracted from satellite images (upper left). The actual cancer prevalence is provided by the CDC 500 Cities project (upper right). A t-SNE was obtained with the extraction features (1). A regularized logistic regression is then fitted with a binned stratified 5-fold cross validation. Out-of-sample predictions were used to plot the map of cancer prevalence in Los Angeles at the census-tract level (2) and the out-of-city generalization was assessed.

All analyses were performed with the pandas (v.0.25.1, Zenodo, Prévessin-Moëns, Switzerland/France) [26], Geopandas (v.0.5.1, Zenodo, Prévessin-Moëns, Switzerland/France) [27], numpy (v.1.17.2, GitHub, Seattle, WA, USA) [28], and Scikit-learn (v.0.21.3, GitHub, Seattle, WA, USA) python 3 libraries [29]. Regression figures were plotted with the matplotlib (v.3.1.1) [30] and seaborn (v.0.9.0, Zenodo, Prévessin-Moëns, Switzerland/France) [31] python 3 libraries. The method is summarized, with Los Angeles as an example, in Figure 3.

## 5. Conclusions

Deep learning was used to extract features from unlabeled satellite images and models were created to correlate these features to cancer prevalence estimates. Using this method, we showed that cancer prevalence can be accurately estimated down to an area of 6000 to 8000 inhabitants. These features are closely correlated to socioeconomic status. Models created on a city can be used on another city with a similar environment to obtain cancer prevalence estimates. This method could potentially be used to assess other health outcomes that are correlated with environment as a new and accurate tool for epidemiological studies.

## Figures and Tables

**Table 1:** Characteristics and results for the cities included in the study (+/−95% confidence interval).

Variable	Chicago	Dallas	Houston	Los Angeles	Phoenix	San Diego	San Jose
Population	2,705,994	1,345,047	2,325,502	3,990,456	1,660,272	1,425,976	1,030,119
Land area (square miles)	588.7	882.90	1651.10	1213.90	1340.60	842.30	459.70
Population density (person/square miles)	4596.56	1523.44	1408.46	3287.30	1238.45	1692.96	2240.85
Number of census	794	304	553	994	356	286	213
Actual cancer prevalence (%)	5.0	4.8	4.8	4.9	5.2	5.0	4.9
*r*^2^ (%)	40.19 (±.23)	41.17 (±20.77)	44.39 (±9.60)	55.27 (±5.28)	64.37 (±6.05)	43.53 (±9.74)	41.54 (±15.23)
Mean squared error	1.17 (±.13)	2.56 (±1.97)	1.36 (±0.28)	1.12 (±0.25)	0.96 (±0.23)	2.30 (±1.27)	1.16 (±0.52)
Mean absolute error	0.81 (±.02)	1.06 (±0.16)	0.88 (±0.10)	0.76 (±0.05)	0.73 (±0.07)	1.03 (±0.14)	0.79 (±0.10)

**Table 2:** *r*^2^ scores of the features extracted from the satellite images to socio-demographic and health population-level measures linked to cancer.

Variable	*r*^2^ Score (%)
Age	78.7
Sex (ref: female)	65.5
Race (ref: non-Hispanic white)	69.7
Education attainment (ref: high school diploma)	72.5
Median per capita income	88.7
Smoking status	89.5
Binge drinking status	85.3
Obesity	91
Diabetes	86.7
Physical activity	89.8

## Supplementary Materials

The following are available online at https://www.mdpi.com/2072-6694/12/12/3844/s1. Figure S1A–G: t-SNE of the cities included in the study; Figure S2A–E: Predicted map of predicted cancer prevalence for the cities included in the study; Figure S3: Scatterplots of model-predicted cancer prevalence in each of the cities. The *y*-axis shows the predicted and the *x*-axis shows the actual cancer prevalence, along with their respective distribution. MSE: mean squared error; Figure S4. Scatterplots illustrating the relationship between the *r*^2^ score (out-of-city model performance) and the cosine similarity. Each dot represents the correlation between the performance of a model trained on one of the ten cities and evaluated on the other nine and the similarity of the satellite images of the city on which the model has been trained and the city on which we evaluated its performance; Table S1: Performances of the different CNN used as feature extractors; Table S2: Characteristics of the convolutional neural network used as a feature extractor.
