# Supplementary material for: Deep Learning Prediction of Cancer Prevalence from Satellite Imagery
Source: Cancers (Basel). 2020 Dec 19;12(12):3844. doi: 10.3390/cancers12123844 (PMC7766226; doi:10.3390/cancers12123844)
Supplement: Supplementary file 1 [file cancers-12-03844-s001.pdf]

cancers

Supplemental Materials

Deep Learning Prediction of Cancer Prevalence from Satellite Imagery

Jean-Emmanuel Bibault, Maxime Bassenne, Hongyi Ren and Lei Xing

Table S1. Performances of the different CNN used as feature extractors.

		Chicago	Dallas	Houston	Los Angeles	Phoenix	San Diego	San Jose	Mean
EfficientNetB7	r^2 (+/- 95% CI)	16,01 (+/- 5,38)	41,73 (+/- 10,21)	31,34 (+/- 6,71)	17,89 (+/- 8,77)	44,73 (+/- 7,9)	6,55 (+/- 6,55)	27,55 (+/- 6,5)	14,761 (+/- 15,1)
	MSE (+/- 95% CI)	1,6 (+/- 0,44)	2,61 (+/- 1,56)	1,54 (+/- 0,3)	1,67 (+/- 0,52)	1,03 (+/- 0,05)	3,31 (+/- 1,4)	1,3 (+/- 0,38)	1,98 (+/- 0,81)
InceptionResnetV2	r^2 (+/- 95% CI)	27,78 (+/- 4,38)	38,45 (+/- 8,92)	34,08 (+/- 8,2)	17,52 (+/- 9,2)	28,17 (+/- 16,2)	14,96 (+/- 6,1)	30,9 (+/- 5,7)	14,824 (+/- 23,22)
	MSE (+/- 95% CI)	1,36 (+/- 0,32)	2,73 (+/- 1,53)	1,48 (+/- 0,35)	1,7 (+/- 0,6)	1,27 (+/- 0,28)	2,95 (+/- 1,2)	1,26 (+/- 0,4)	1,966 (+/- 0,76)
NASNetLarge	r^2 (+/- 95% CI)	26,26 (+/- 4,49)	38,7 (+/- 7,25)	30,05 (+/- 6,75)	21,72 (+/- 6,31)	26,19 (+/- 13,6)	7,49 (+/- 10,7)	15,3 (+/- 8,3)	12,45 (+/- 21,77)
	MSE (+/- 95% CI)	1,39 (+/- 0,34)	2,68 (+/- 1,35)	1,6 (+/- 0,39)	1,64 (+/- 0,6)	1,34 (+/- 0,13)	3,11 (+/- 1,2)	1,53 (+/- 0,44)	2,013 (+/- 0,75)
ResNet50	r^2 (+/- 95% CI)	35,06 (+/- 4,86)	38,93 (+/- 12,71)	33,02 (+/- 6)	21,12 (+/- 7,98)	35,55 (+/- 11,8)	14.46 (+/- 4,8)	31,6 (+/- 6)	15,64 (+/- 25,56)
	MSE (+/- 95% CI)	1,22 (+/- 0,3)	2,79 (+/- 1,95)	1,51 (+/- 0,29)	1,66 (+/- 0,67)	1,16 (+/- 0,1)	3,01 (+/- 1,3)	1,25 (+/- 0,4)	1,952 (+/- 0,78)
VGG16	r^2 (+/- 95% CI)	25,31 (+/- 4,03)	37,02 (+/- 8,78)	23,86 (+/- 6,08)	17,56 (+/- 7,8)	40,4 (+/- 10,5)	-6,32 (+/- 8,9)	18,56 (+/- 10)	11,548 (+/- 22,45)
	MSE (+/- 95% CI)	1,43 (+/- 0,44)	2,78 (+/- 1,52)	1,72 (+/- 0,32)	1,72 (+/- 0,62)	1,09 (+/- 0,11)	3,62 (+/- 1,3)	1,46 (+/- 0,43)	2,055 (+/- 0,51)

Table S2. Characteristics of the convolutional neural network used as feature extractor.

	ResNet50
Depth	176
Number of parameters	23,534,592
Number of features generated	2,048
Input shape	(224, 224, 3)

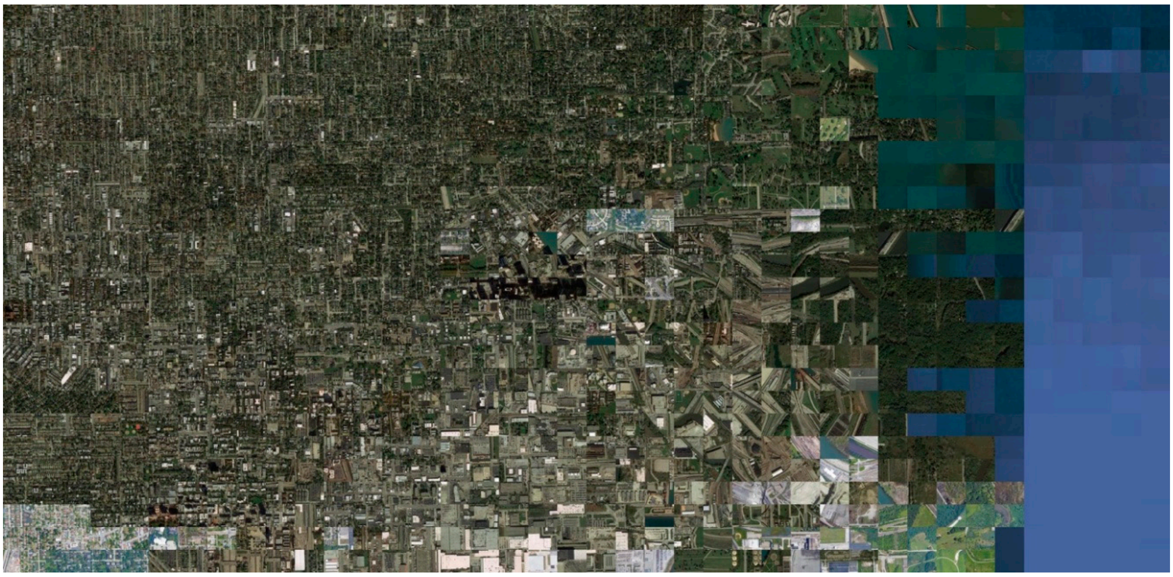

Figure S1A. t-SNE of a random subsample of 1000 images from Chicago, IL.

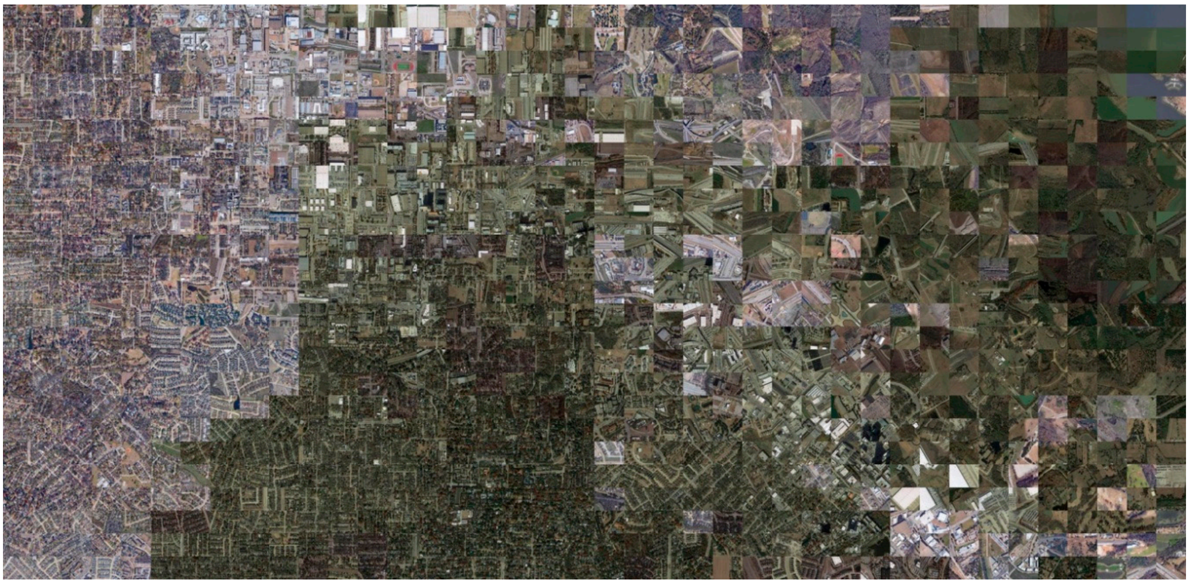

Figure S1B. t-SNE of a random subsample of 1000 images from Dallas, TX.

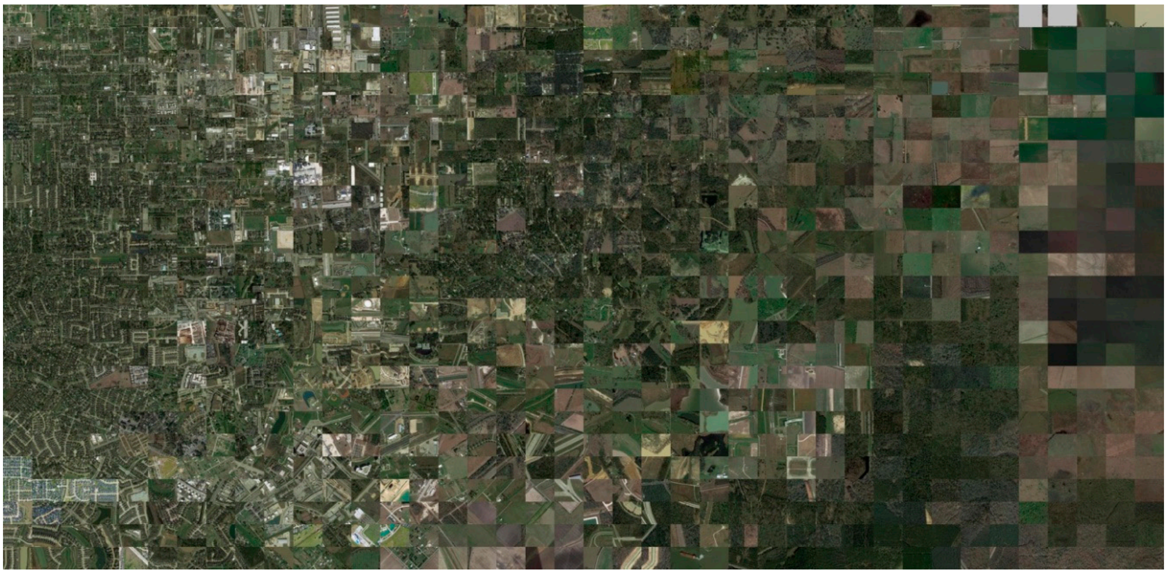

Figure S1C. t-SNE of a random subsample of 1000 images from Houston, TX.

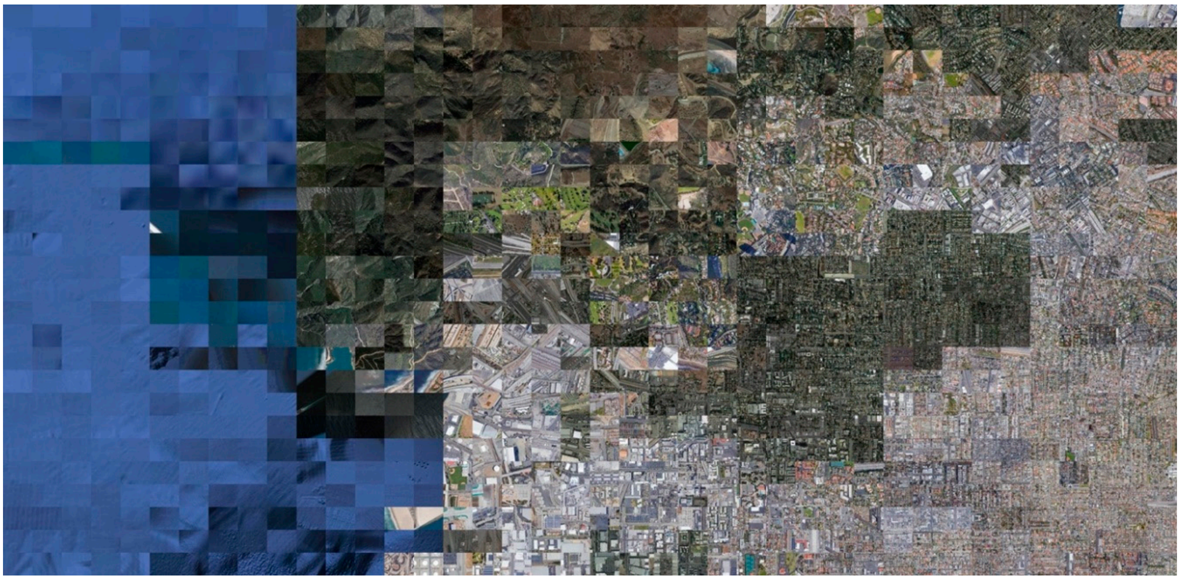

Figure S1D. t-SNE of a random subsample of 1000 images from Los Angeles, CA.

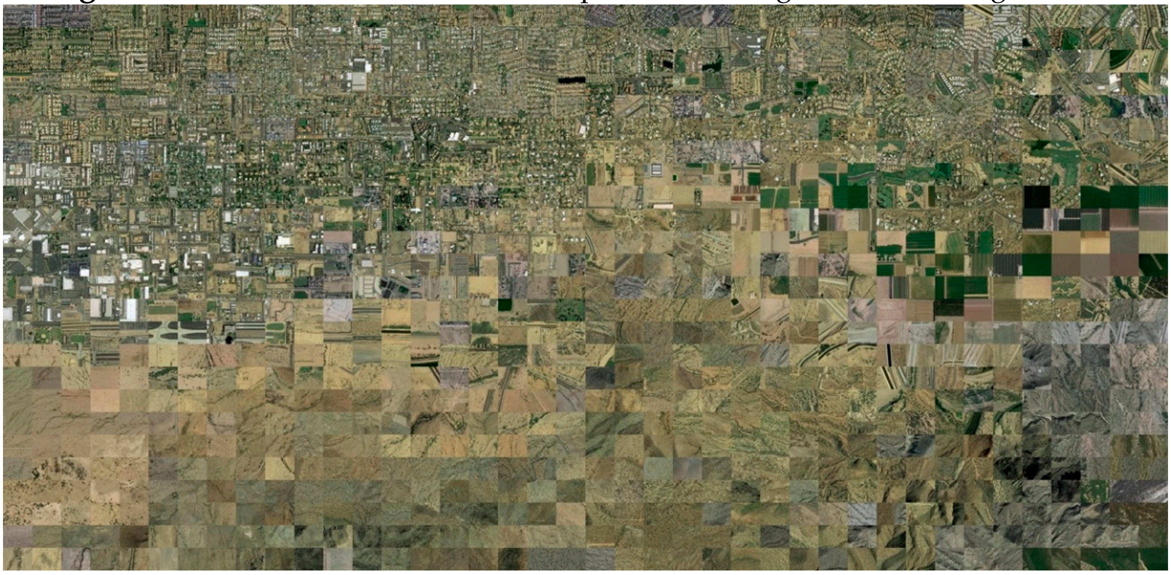

Figure S1E. t-SNE of a random subsample of 1000 images from Phoenix, AZ.

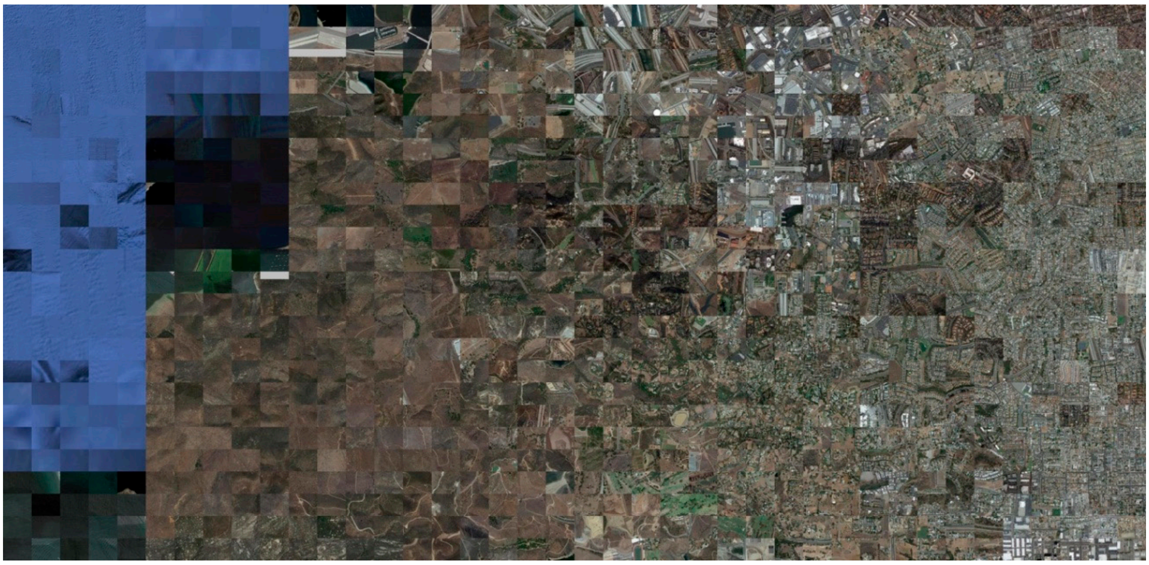

Figure S1F. t-SNE of a random subsample of 1000 images from San Diego, CA.

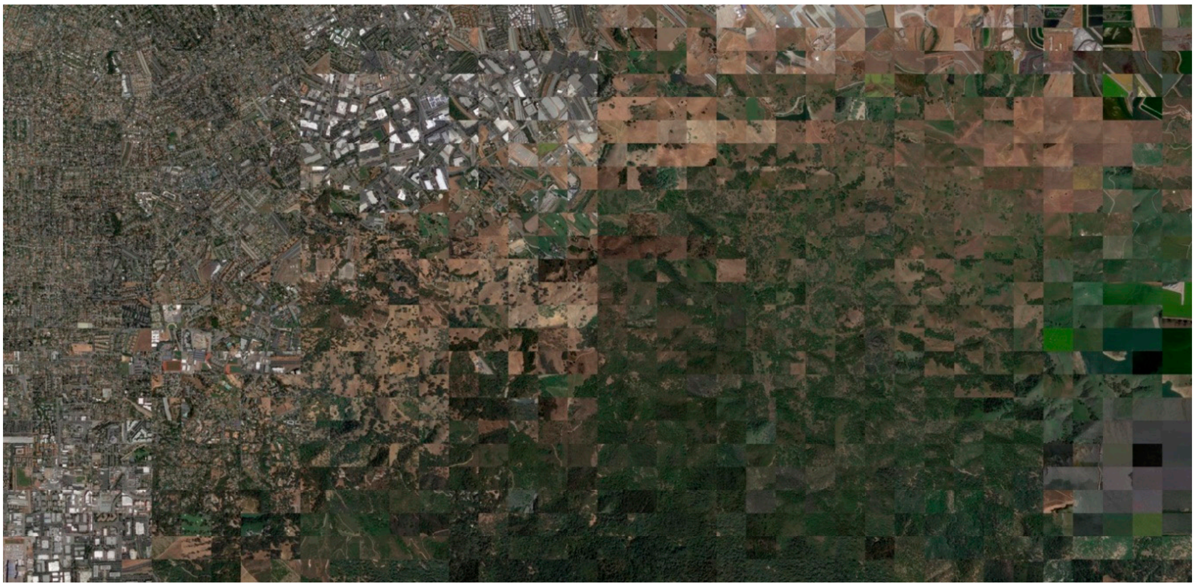

Figure S1G. t-SNE of a random subsample of 1000 images from San Jose, CA.

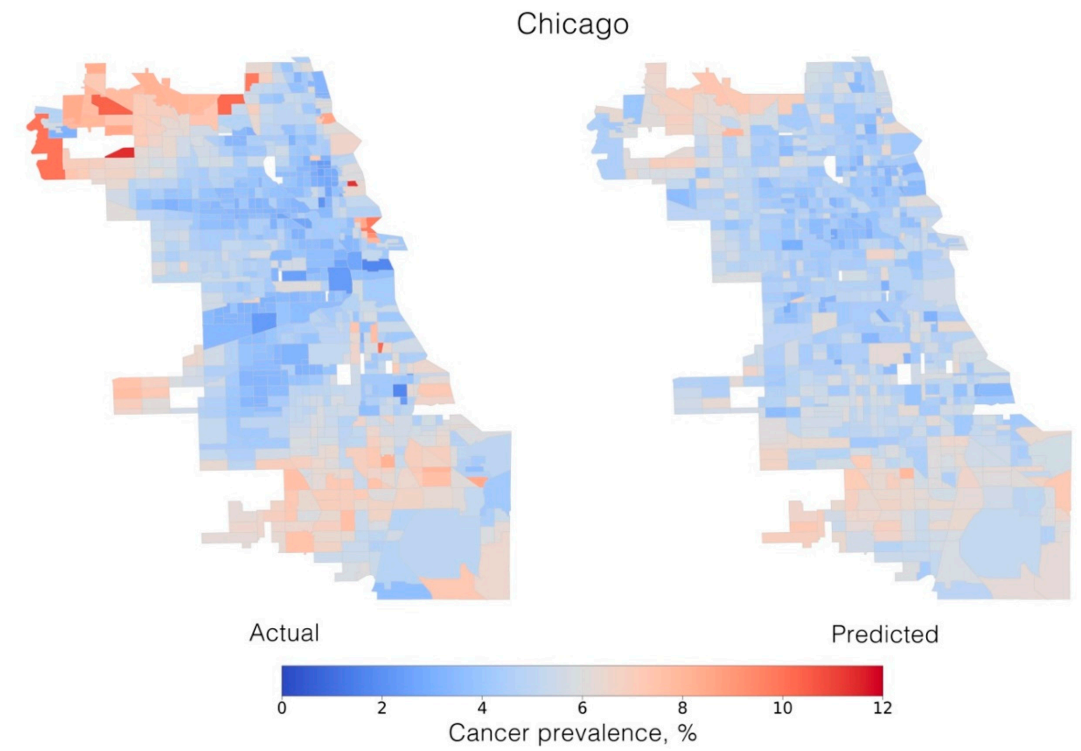

Figure S2A. Maps of actual (left) and predicted (right) cancer prevalence in Chicago, IL at the census-tract level.

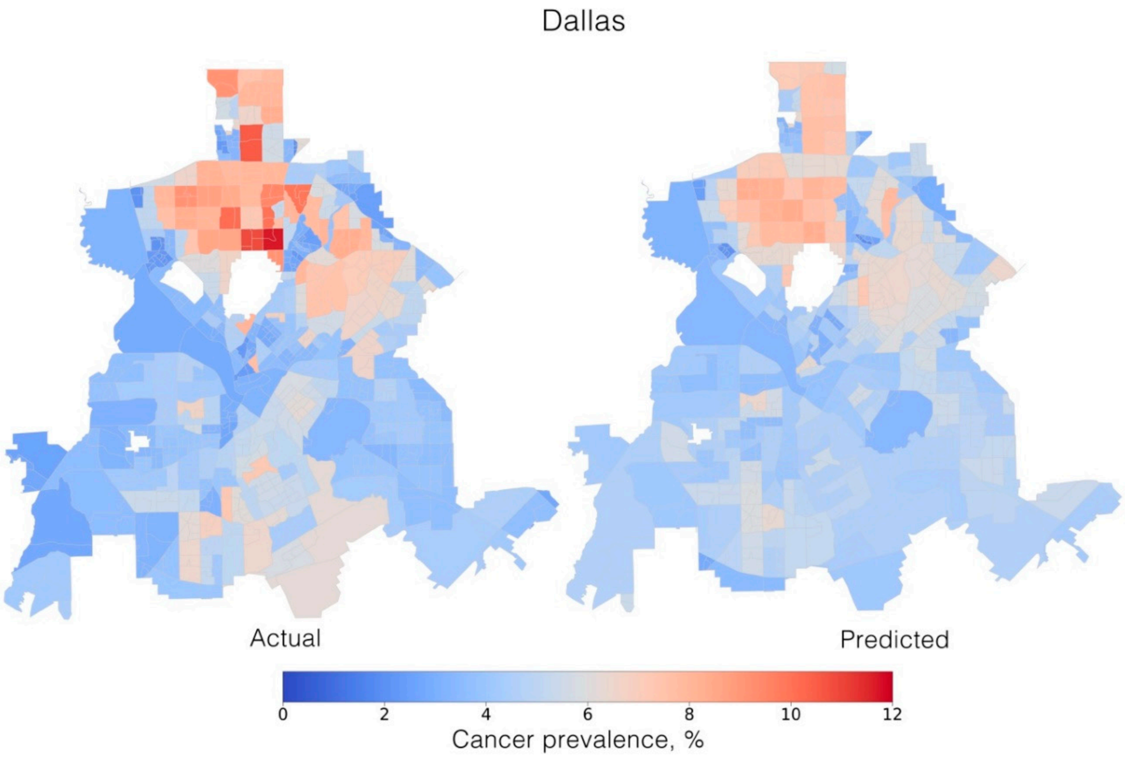

Figure S2B. Maps of actual (left) and predicted (right) cancer prevalence in Dallas, TX at the census-tract level.

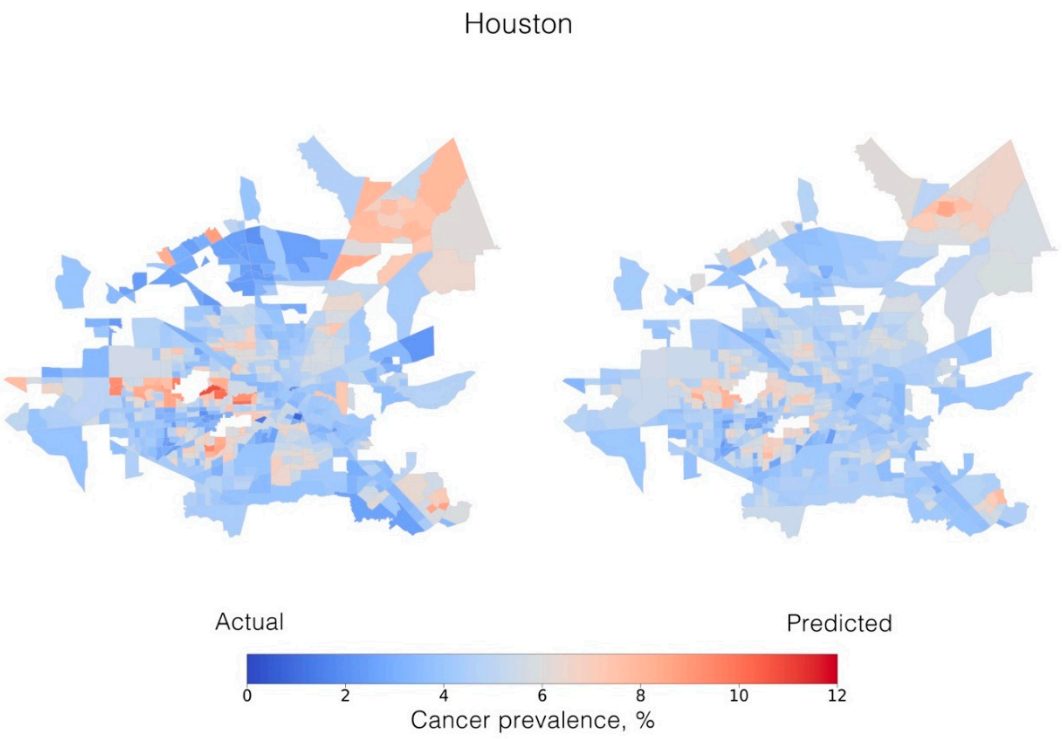

Figure S2C. Maps of actual (left) and predicted (right) cancer prevalence in Houston, TX at the census-tract level.

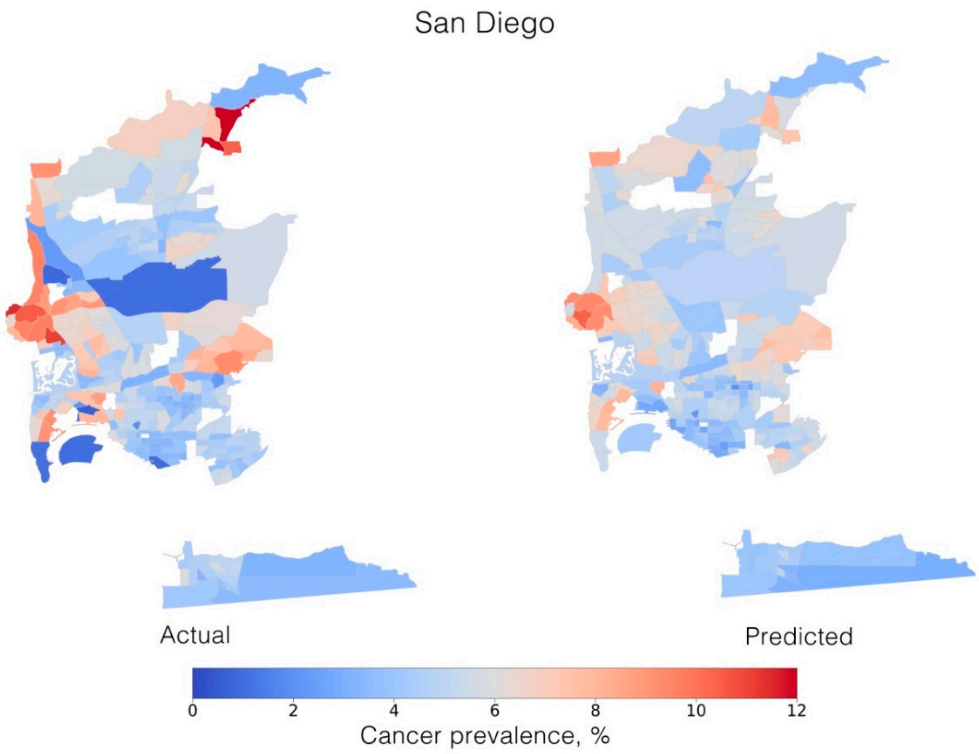

Figure S2D. Maps of actual (left) and predicted (right) cancer prevalence in San Diego, CA at the census-tract level.

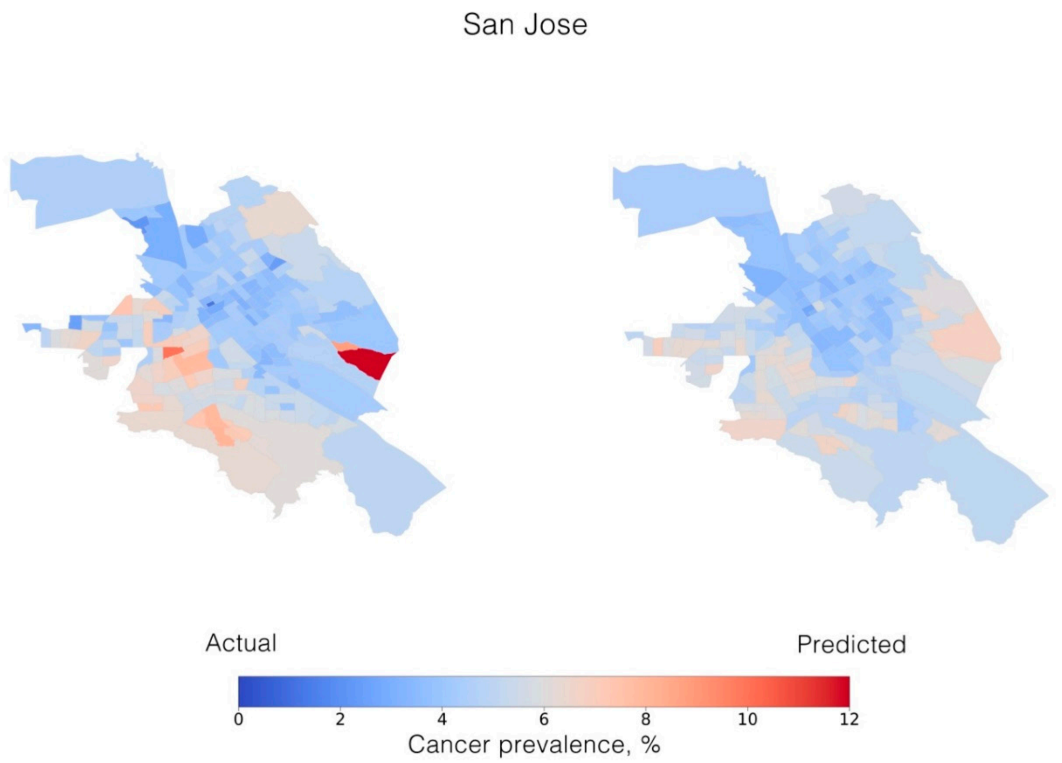

Figure S2E. Maps of actual (left) and predicted (right) cancer prevalence in San Jose, CA at the census-tract level.

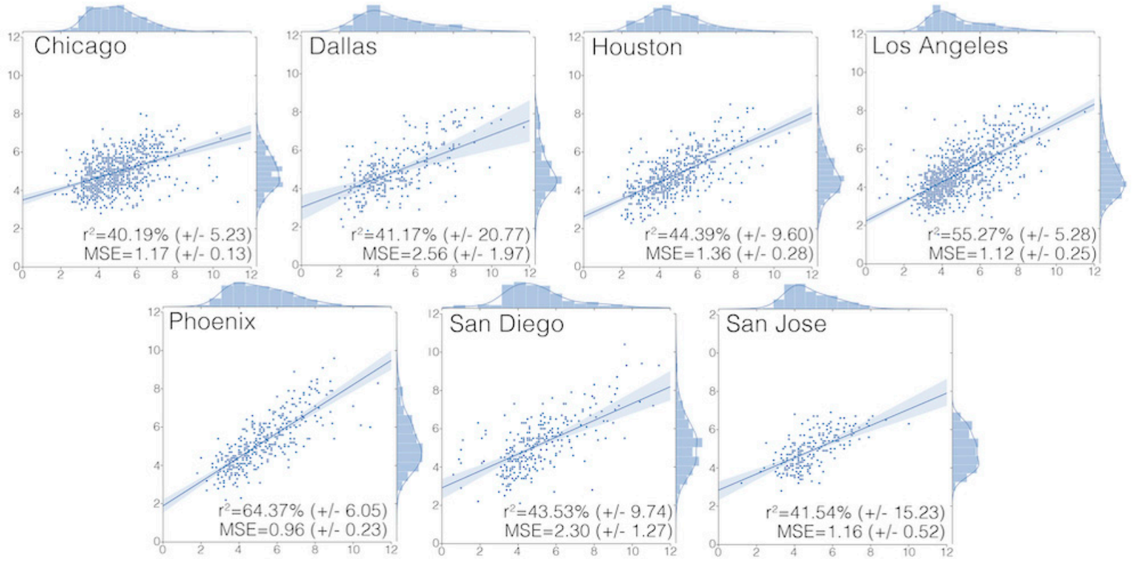

Figure S3. Scatterplots of model-predicted cancer prevalence in each of the cities. The y-axis shows the predicted and the x-axis shows the actual cancer prevalence, along with their respective distribution. MSE: mean squared error.

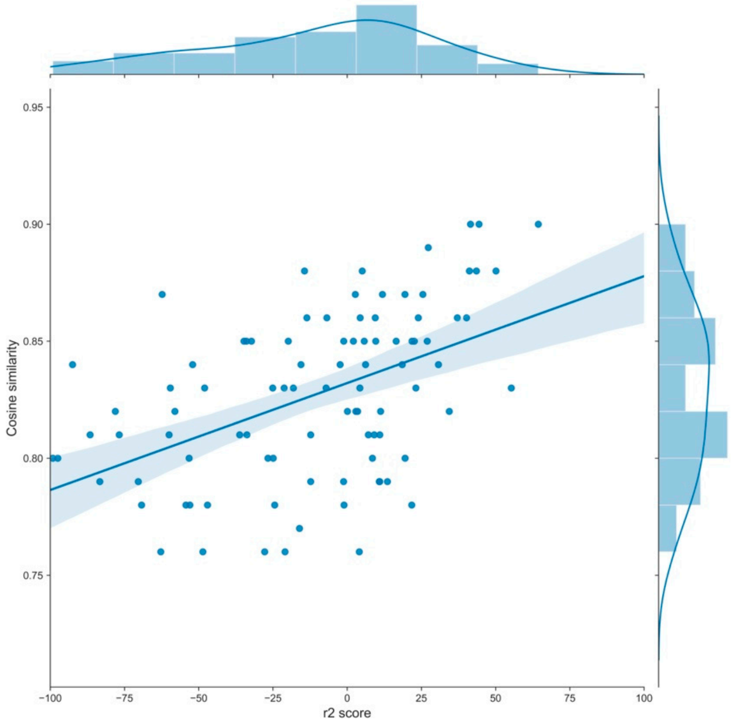

Figure S4. Scatterplots illustrating the relationship between r2 score (out-of-city model performances) and the cosine similarity. Each dot represents the correlation between the performance of a model trained on one of the ten cities and evaluated on the other nine and the similarity of the satellite images of the city on which the model has been trained and the city on which we evaluate its performance.
